# Evaluation of anti-Fel d 1 IgY ingredient for pet food on growth performance in kittens

**DOI:** 10.3389/fvets.2024.1355390

**Published:** 2024-03-05

**Authors:** Erik D. Hedrick, Ray A. Matulka, Lisa Conboy-Schmidt, Kimberly A. May

**Affiliations:** ^1^Burdock Group Consultants, Orlando, FL, United States; ^2^Nestlé Purina PetCare Global Resources, Vevey, Switzerland

**Keywords:** Fel d 1 IgY, feline, safety, cat, Fel d 1, allergen, kitten

## Abstract

**Introduction:**

The domestic cat (Felis catus) is one of the most common pets. Worldwide, approximately one in five adults are sensitive to cat allergens. The major cat allergen is the secretoglobulin Fel d 1, which is primarily produced in the salivary and sebaceous glands. Chickens produce IgY antibodies, which are similar in structure to mammalian IgG. When chickens are exposed to Fel d 1, anti-Fel d 1-specific IgY (AFD1) is produced and is naturally concentrated in egg yolk. The aim of this study was to evaluate the tolerability, effects on growth and food consumption, and potential adverse effects of a chicken egg product ingredient containing AFD1 in kittens.

**Methods:**

This was a blinded, controlled study. Twenty-seven (27) eight-week old kittens were randomly assigned to three feeding groups containing 0 ppm AFD1 (Group 0), 8 ppm AFD1 (Group 1), and 16 ppm AFD1 (Group 2) for 84 days. Veterinary exams and bloodwork were performed on Day 42 and Day 84, and body weight and body condition score (BCS) were monitored weekly.

**Results:**

Throughout the study, there were no signs of nutritional deficiency or adverse clinical events in any of the subjects. Administration of a chicken egg product ingredient containing AFD1 in the diet (whether in coating or combination of coating and top dress) had no significant effect on body weight nor food consumption, and all subjects maintained a healthy Body Condition Score (BCS) throughout the study. Moreover, there were no biologically significant differences in the mean clinical chemistry and hematology parameters.

**Discussion:**

This study demonstrated that a diet formulated to contain up to 16  ppm AFD1, included in the coating and the top-dress of dry kitten food, was well tolerated, promoted adequate growth, and exhibited no adverse effects.

## Introduction

Cats are the second most common pet in the United States and worldwide. Cat allergies are the most common mammalian-derived allergy in humans, affecting approximately 1 in 5 adults worldwide (1, 2). Cats produce multiple allergens, and, to date, eight cat-specific allergens that elicit a specific IgE- or IgG-mediated allergic response have been documented (3, 4). Fel d 1 is the major cat allergen, accounting for up to 96% of human allergic sensitization to cats and 60–90% of the overall antigenicity of cats and their dander (3, 5). The allergen is primarily produced in a cat’s sebaceous and salivary glands (3, 5, 6). Salivary-origin Fel d 1 is transferred from saliva to cats’ hair when cats groom themselves and transferred to the environment on shed hair and dander. All cats produce Fel d 1 regardless of breed, age, hair (length, color or pattern), sex (male or female; neutered or intact), housing (indoors vs. outdoors), or body weight; there are no truly allergen-free or hypoallergenic cats (3, 5–10). Fel d 1’s function for the cat has not been definitively determined, but proposed roles include pheromone/chemical signaling, epithelial defense, lipid regulation and immunoregulation (11–18).

Fel d 1 triggers an IgE-mediated allergic reaction in sensitized individuals. Symptoms of human allergic response to Fel d 1 range from mild rhinoconjunctivitis to severe anaphylactic reactions, including asthmatic exacerbations (19). Humans are exposed to the Fel d 1 antigen present on cat hair and in the environment; the allergen readily becomes airborne and can adhere to fabrics, carpet, and upholstery (17, 20–22). Fel d 1 may also be passively transported on clothing and portable items; as a result, the allergen is ubiquitous and has been documented in private vehicles, and public transportation and buildings at levels that exceed the threshold value associated with sensitization and may worsen allergic symptoms in sensitized individuals (3, 23–31). Considerable resources have been dedicated to developing new ways to improve the relationship between domesticated cats and Fel d 1-sensitized people.

The chicken (*Gallus gallus domesticus*) has been a source of nutrition for man and other carnivores for over three millennia by means of its meat and eggs (32, 33). Eggs contain complex nutrients and components that are essential for the development of the chick embryo, including immunologically active immunoglobulin Y (IgY) (34, 35). It has been demonstrated that IgY can be beneficial for other animals and has been added to commercially prepared animal feed for decades (36–39). In chickens, IgY has similar structural and functional properties as mammalian IgG (38, 40). When exposed to Fel d 1, chickens naturally produce anti-Fel d 1-specific IgY (AFD1). Egg yolk powder containing AFD1 can bind and neutralize the Fel d 1 antibody in the cat’s saliva (41–43). Neutralized Fel d 1 is unable to bind to IgE and therefore cannot induce allergic responses in sensitized humans.

New ingredients or ingredients with novel characteristics must undergo stringent safety studies that demonstrate safety under the intended conditions of use prior to commercial release as pet food (44). Recently, it has been demonstrated that a chicken egg product containing AFD1 is well tolerated, had no adverse health effects, and did not induce any clinically significant alterations in laboratory parameters in adult cats and did not exhibit genotoxicity or mutagenicity potential *in vitro* (33). The kitten life stage is considered to be under 1 year of age (45). This study was conducted to evaluate the effect of the addition of AFD1 egg product to a complete and balanced kitten diet on growth performance in 27 kittens when compared to a control kitten diet for 12 weeks (until kittens were approximately 21 weeks of age). The diets were formulated to provide 0, 8, or 16 ppm AFD1. Other parameters such as food consumption, clinical and hematological parameters, and taurine levels were also analyzed to assess the effects of the AFD1 diet.

## Materials and methods

### Study design

This was a randomized, blinded investigative study conducted using a parallel matched-group design.

### Test feeding ingredient

An egg product ingredient containing IgY immunoglobulins specific for Fel d 1 antigen was provided by the Nestlé Purina PetCare Company. The egg product ingredient was an offwhite, granular processed egg yolk powder with a maximum 5% moisture, greater than 28% protein and a maximum 7% ash, providing at least 1,000 parts per million (ppm) anti-fel d1 IgY. This ingredient was applied as a coating on the test diet and incorporated into a top-dress.

### Acclimation period

Twenty-eight weaned kittens were obtained at 8 weeks (±4 days) of age at study initiation. All kittens remained under quarantine for 1 week (study days −7 to −1), during which initial veterinary examinations, body weight measurements, and fecal analysis, as well as blood collections for hematology and clinical chemistry analysis, were performed. This period also allowed for transition to the control diet (a commercially available complete and balanced kitten diet) as well as acclimation to the study conditions prior to initiation of the study. During the quarantine/acclimation period, twice daily health observations and food consumption measurement were performed. All kittens were treated for coccidia with the anthelminthic toltrazuril (20 mg/kg PO q24) for 3 days, beginning on Day −1 and ending on Day 1; fecal samples obtained 2 weeks later confirmed successful resolution. The kittens received a multivalent feline viral rhinotracheitis, feline calicivirus, and feline panleukopenia (FVRCP) vaccine at 8, 12, and 16 weeks of age (Day −6, 21, and 49, respectively, of study). No other medications were administered over the course of the study. General health observations were conducted twice daily over the course of the study. Kittens were observed for any signs that would not be expected in healthy cats.

### Allocation/randomization and diet trial

Kittens that met the following criteria were included in the study: (1) were 8 weeks of age (±4 days) at the time of arrival to the test facility; (2) were eating solid food; (3) had a cooperative disposition; (4) did not require medications or supplements that could interfere with the study objectives; and (5) were deemed to be in good general health by the facility veterinarian as determined by baseline veterinary examinations, hematology, and clinical chemistry analysis.

Twenty-seven (14 males and 13 females) kittens from the larger group of 28 were selected to continue to the test phase based on health and behavior. The excluded kitten had the lowest body weight and showed the lowest food consumption. Each of the remaining kittens was allocated to 1 of the 3 groups (Groups 0, 1, and 2) using a random number generator until there were nine kittens in each group. Males and females were allocated separately so the ratio of males to females was similar between groups to the extent possible. Each kitten was given a name and a tattoo for identification purposes.

Groups were then randomly assigned to one of three diets (0, 8 and 16 ppm AFD1), designated by color for blinding, and cats in each group were fed a combination of a complete and balanced kitten dry diet and a top-dress (Table 1). The control diet (Group 0; 0 ppm AFD1) was a complete and balanced kitten food to which control top dress egg yolk powder was added. The test diets were the control diet coated with an egg product providing a total of 8 ppm (Group 1) or 16 ppm AFD1 (Group 2). The control top-dress and test top-dress differed only in their inclusion of egg powder containing AFD1; the control top-dress included egg yolk powder without AFD1, whereas the test top-dress contained egg yolk powder with AFD1. The top-dress was applied immediately prior to feeding. The test ingredient was an off-white, granular processed egg yolk powder with a maximum 5% moisture, greater than 28% protein and a maximum 7% ash.

**Table 1:** Test group summary.

Group	Formulated total anti-fel d1 IgY (AFD1) (ppm)	Diet description
Group 0 (control)	0	Complete and balanced kitten food with control top-dress
Group 1 (test 8 ppm)	8	Complete and balanced kitten food with test coating and control top-dress
Group 2 (test 16 ppm)	16	Complete and balanced kitten food with test coating and test top dress

**Figure 1:**
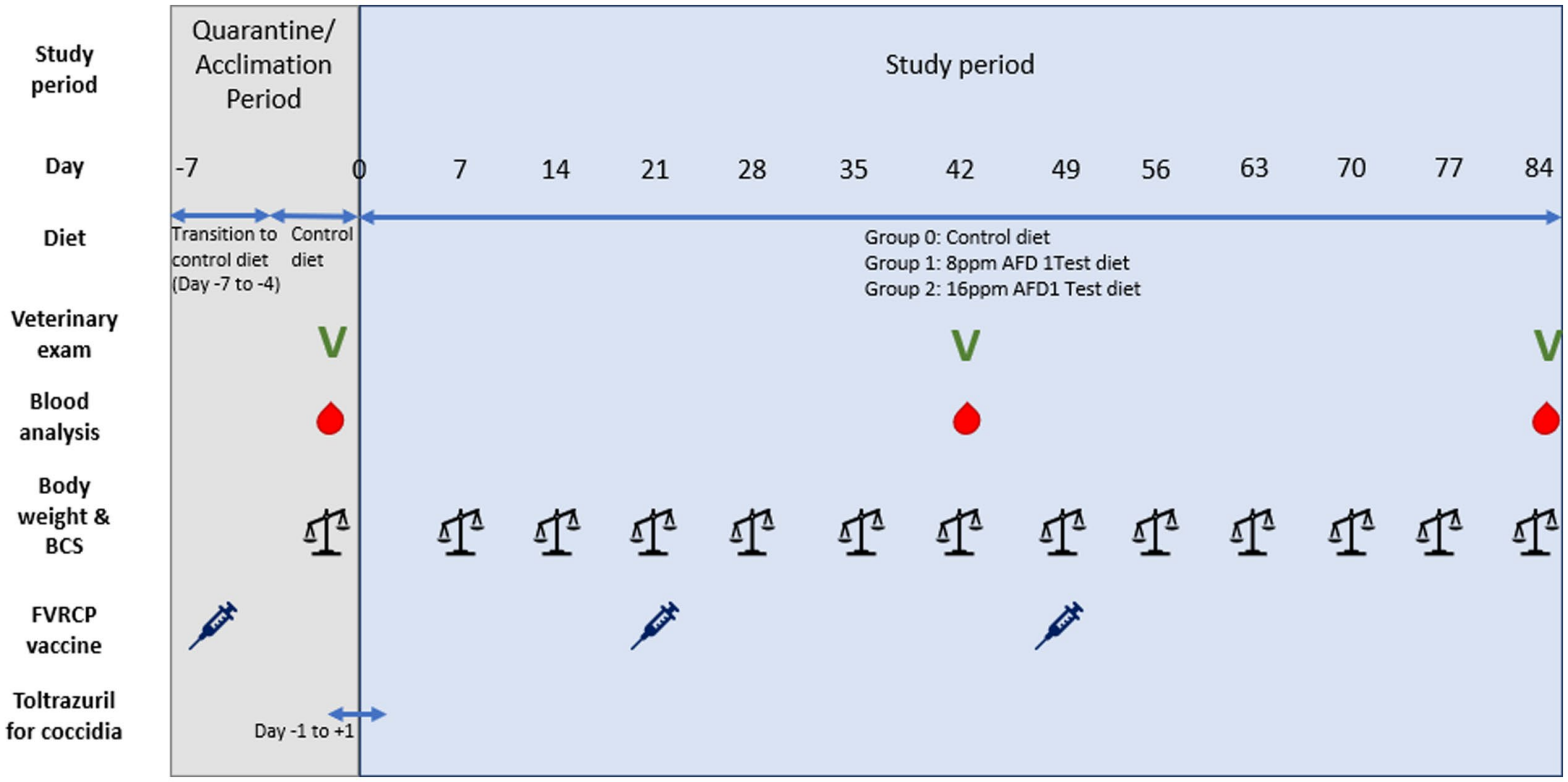
Graphic timeline of the study.

Kittens remained on their assigned diets for through Day 84 of the study (until approximately 21 weeks of age). Figure 1 provides a graphic representation of the study timeline.

### Blinding

Diets were received with full labeling, with each being assigned a group label (Group 0, 1, or 2) by unblinded technicians. The group label was used as the test identifier over the course of the study. The study was blinded to the technicians who administered the diet to the kittens as well as the personnel responsible for performing group allocation and the technicians and investigators collecting samples and recording data. Only information pertinent to diet administration was recorded by unblinded technicians.

### Animal welfare statement and justification for use of test animals

Procedures were designed to avoid or minimize discomfort, distress, and pain to the animals in accordance with the principles of The Animals for Research Act of Ontario and the guidelines of Canadian Council on Animal Care (CCAC). The CCAC Guide for the Care and Use of Experimental Animals and related policies were regarded as guidelines to follow. To ensure compliance, this protocol was reviewed and approved by CanCog (Vivocore Internal Animal Care Committee) before the start of the study. Cats were the target species for the investigational diet under evaluation. The number of animals involved in this study was the minimum required by the Association of American Feed Control Officials (AAFCO) for feeding protocols to support a growth claim for a cat food (46).

### Housing conditions, husbandry, and veterinary care

Kittens were group-housed in a single containment room separated from the rest of the facility colony; however, the kittens were individually housed for feeding procedures to ensure consumption of only their feeding allotment and assigned diet. Housing was carried out according to the recommendations of the Canadian Council on Animal Care. All animal housing areas were cleaned daily and disinfected according to the testing facility’s standard operating procedures. Upon arrival to the test facility, all kittens were subject to isolation in a separate housing area for biosecurity purposes. Disease transmission prevention practices were implemented, and cross contamination activities avoided according to standard operating procedures.

A combination of LED artificial lighting and natural light was provided for the kittens. The photoperiod for the artificial lighting was approximately 12 h (7:00 to 19:00, maintained on a timer); however, natural light was provided without restriction. Environmental temperature was provided by radiant floor heating and a forced air system, electronically controlled and set to maintain the animal housing rooms between 18.6°C and 25.9°C. The range of humidity for the housing room was 32–74% over the course of the study. The housing room ventilation was set for negative pressure and was controlled by air exchanger and set at a level required to maintain the environmental temperature levels and allow for fresh air to be supplied continuously at an exchange rate of 18 air exchanges *per* hour.

Weekly body weight measurements, twice daily general health observations, and twice daily food consumption measurement, as well as handling and socialization exposure four times weekly, were performed over the course of the study. Body weight measurements were performed using a certified, verified scale according to the facility’s standard operating procedures. At the time of weight measurement, kittens were also given a body condition score using the Purina 9-point scale, with 5/9 being the ideal body condition (range, 1/9 emaciated and 9/9 obese).

Veterinary examinations were performed on Day −6 (the day following arrival to the test facility), Day 42 (study midpoint), and Day 84 (study conclusion). Examinations included an assessment of general health, hair coat and skin, ears, mouth, nose, throat, musculoskeletal system, eyes, abdomen (including mammary chain examination and external abdominal palpation), external urogenital system, nervous system, temperature, and behavior as well as auscultation of the heart, trachea and lungs.

### Feeding and watering

All diets and top-dresses were manufactured and supplied by the Nestlé Purina PetCare Company. Unopened bags of diet and top-dress were stored in a secure, climate-controlled storage room with minimal exposure to light. Opened bags were stored in airtight containers labeled with the study number and Group code. A minimum 100-g food sample and a minimum 1-g top-dress sample were collected from each opened bag and frozen at approximately −20°C for later analysis. Samples were shipped on ice to the Nestlé Purina PetCare Company at study conclusion.

Each kitten’s daily allotment of food was divided into two equal feedings (morning and evening). Upon arrival to the test facility, the kittens were transitioned from the breeding facility diet to the control diet. From Day −7 to Day −5, daily rations consisted of 25, 50, and 75% of the control diet, respectively, until the kittens were fully transitioned to the control diet by Day −4. Beginning on Day 0 and continuing until study completion, kittens received their assigned group diet and corresponding top-dress. Kittens were individually fed for the duration of the study to maintain body condition scores of 5 on the Purina 9-point scale. Initially, kittens were fed 30 g of kibble (equivalent to 0.25 cups); however, amounts were adjusted as necessary based on recommendations from the facility veterinarian.

Application of top-dress to food was performed at the time of feeding. Top-dress amounts (0.5 g *per* feed offering, containing approximately 0 or 8 ppm AFD1 based on group) were weighed using a certified, verified scale according to standard operating procedures. Top-dress for each group was weighed separately, and utensils were cleaned between groups to avoid cross contamination. Initially, top-dress was mixed with each food ration until it was evenly distributed over the kibble; however, following reports of top-dress residue present on leftover food, mixing the top-dress into the food was discontinued after Day 6. Beginning on Day 7 of the study, the top-dress was evenly distributed on top of the kibble to increase the likelihood of kittens ingesting the entire intended dose of the top-dress. Water was provided *ad libitum* in stainless steel bowls. Water consumption was not determined over the course of the study.

### Blood collections for hematology and clinical chemistry analysis

Whole blood collections for hematology and clinical chemistry analysis were performed on Day −6 (baseline) and on Day 84 (study conclusion). The kittens were fasted for a minimum of 4 h prior to the baseline blood collection and the final blood collection on Day 84. At each time point, approximately 1 mL of venous blood was collected from the right or left jugular vein. From each sample, a minimum of 600 μL was transferred into a K2EDTA tube and inverted gently to ensure proper mixing of tube additives. Tubes were placed on wet ice until refrigeration at 2–8°C. The remaining 400 μL was transferred into a serum separator tube and inverted gently. Serum tubes were allowed to clot at room temperature for 10 min prior to centrifugation at 1,525 to 1,992 rcf for 10 min at ambient temperature. Serum tubes were then refrigerated at 2–8°C. On the same day as collection, samples were shipped on wet ice to IDEXX Bioanalytics for overnight analysis.

The following clinical chemistry parameters were evaluated: alkaline phosphatase (ALP), aspartate transaminase (AST), alanine transaminase (ALT), albumin (ALB), total bilirubin, total protein, globulin (GLOB), blood urea nitrogen (BUN), creatinine, cholesterol, glucose, calcium, phosphorus, chloride, potassium, ALB/GLOB ratio, sodium, and the BUN/creatinine ratio. The following hematological parameters were evaluated: hemolysis, lipemia, white blood cell (WBC) count, red blood cell (RBC; erythrocyte) count, hemoglobin (HGB), hematocrit (HCT), mean corpuscular volume (MCV), mean corpuscular hemoglobin (MCH), mean corpuscular hemoglobin concentration (MCHC), platelet count, reticulocyte percent, absolute reticulocytes, nucleated RBS, Heinz bodies, neutrophils, lymphocytes, monocytes, eosinophils, and basophils (percentage and number).

### Blood collections for taurine analysis

On Day 84, at the time of blood collection for hematology and clinical chemistry analysis, an additional 0.5–1 mL of blood was obtained for the purpose of taurine analysis. The sample was transferred to a lithium heparin tube, inverted gently to ensure proper mixing of tube additives, and placed on wet ice until refrigerated at 2–8°C. On the day of collection, the lithium heparin tubes containing whole blood were then shipped on wet ice to IDEXX Bioanalytics where they were processed by centrifugation to isolate plasma. Isolated plasma was then transferred to no-additive tubes and shipped the same day on wet ice to the University of California, Davis where the plasma taurine analysis was performed. Sample processing methods for taurine in plasma were as follows: samples were hydrolyzed using 6 Nmol HCl in a sealed ampoule (110°C, 24 h), dried with nitrogen gas, dissolved again in loading buffer, filtered, and loaded in on a Biochrom 30 amino acid analyzer without dilution.

### Data analysis

Data analyses for group differences in body weight and food consumption were conducted utilizing repeated measures ANOVA analysis. Hematological and clinical chemistry parameters were assessed for normality using the Kolmogorov–Smirnov Test. For the parameters in which the normality assumption could not be rejected, analysis was performed utilizing a one-way ANOVA analysis. Taurine levels between groups were analyzed using a main effects ANOVA. Groups that showed significant differences between feeding groups underwent post-hoc analysis using the Tukey’s multiple comparison test.

## Results

### Growth and food consumption

**Figure 2:**
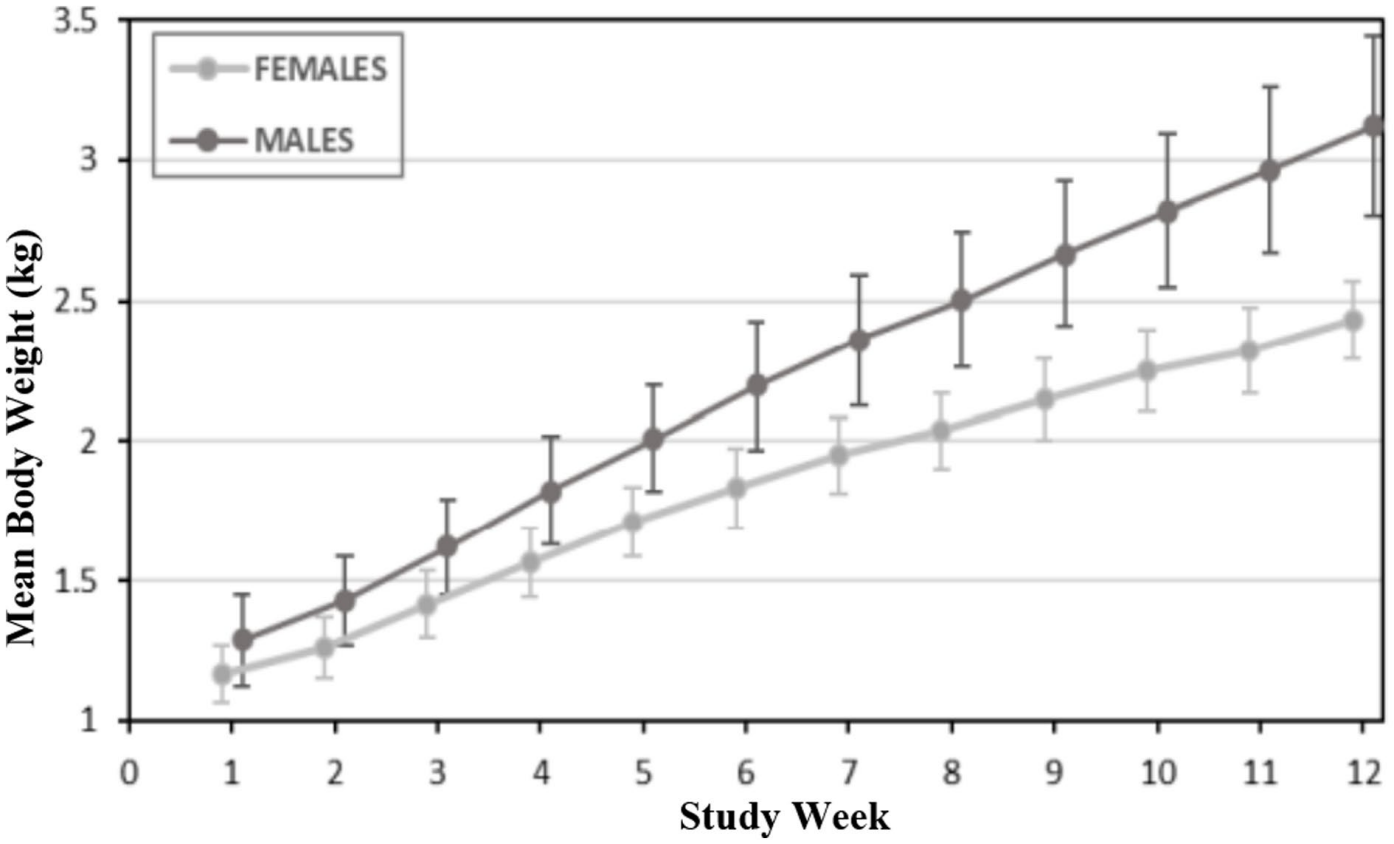
Mean body weights (kg) for male and female kittens.

**Figure 3:**
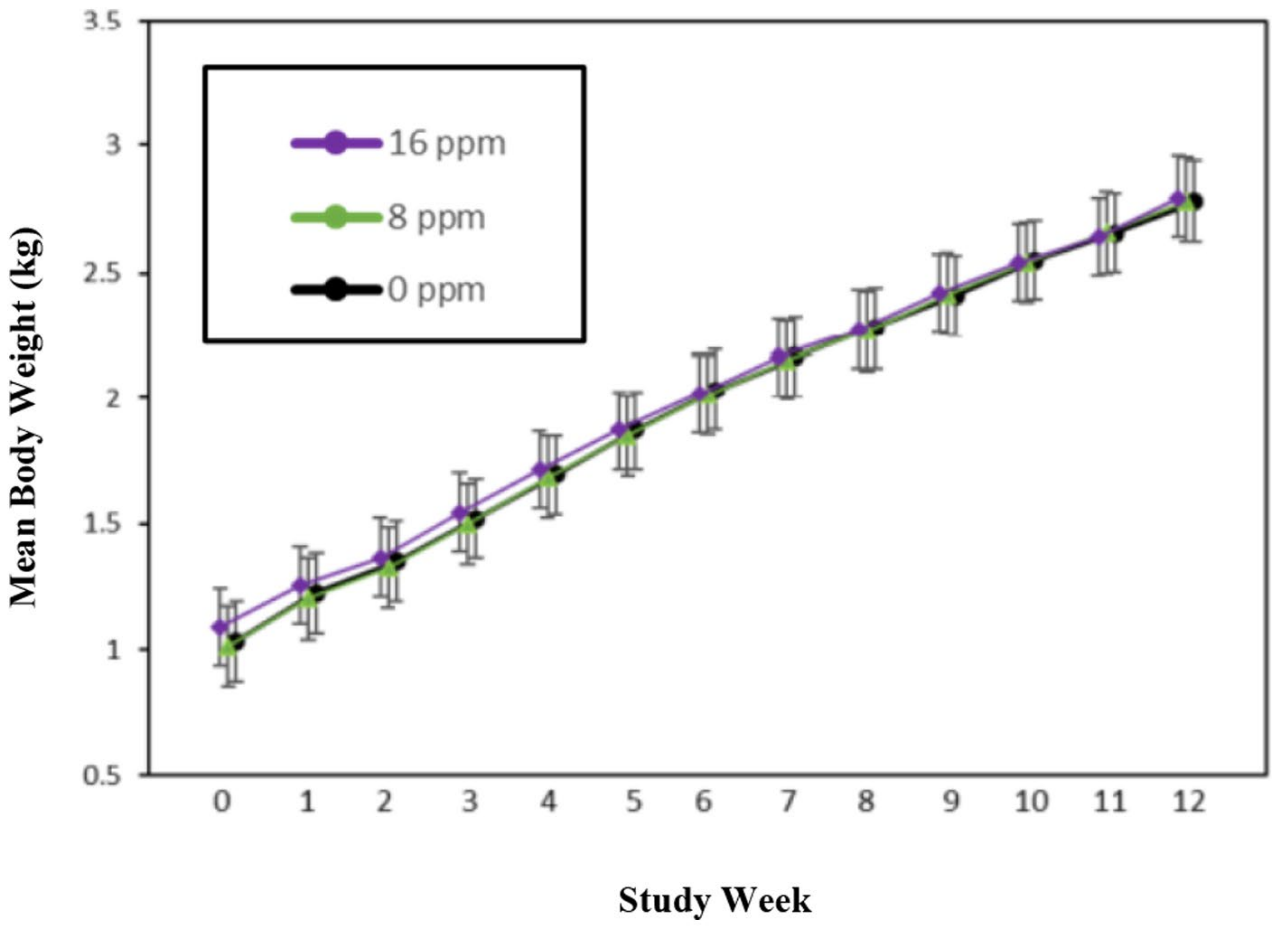
Mean body weights (kg) for the three diet groups.

**Figure 4:**
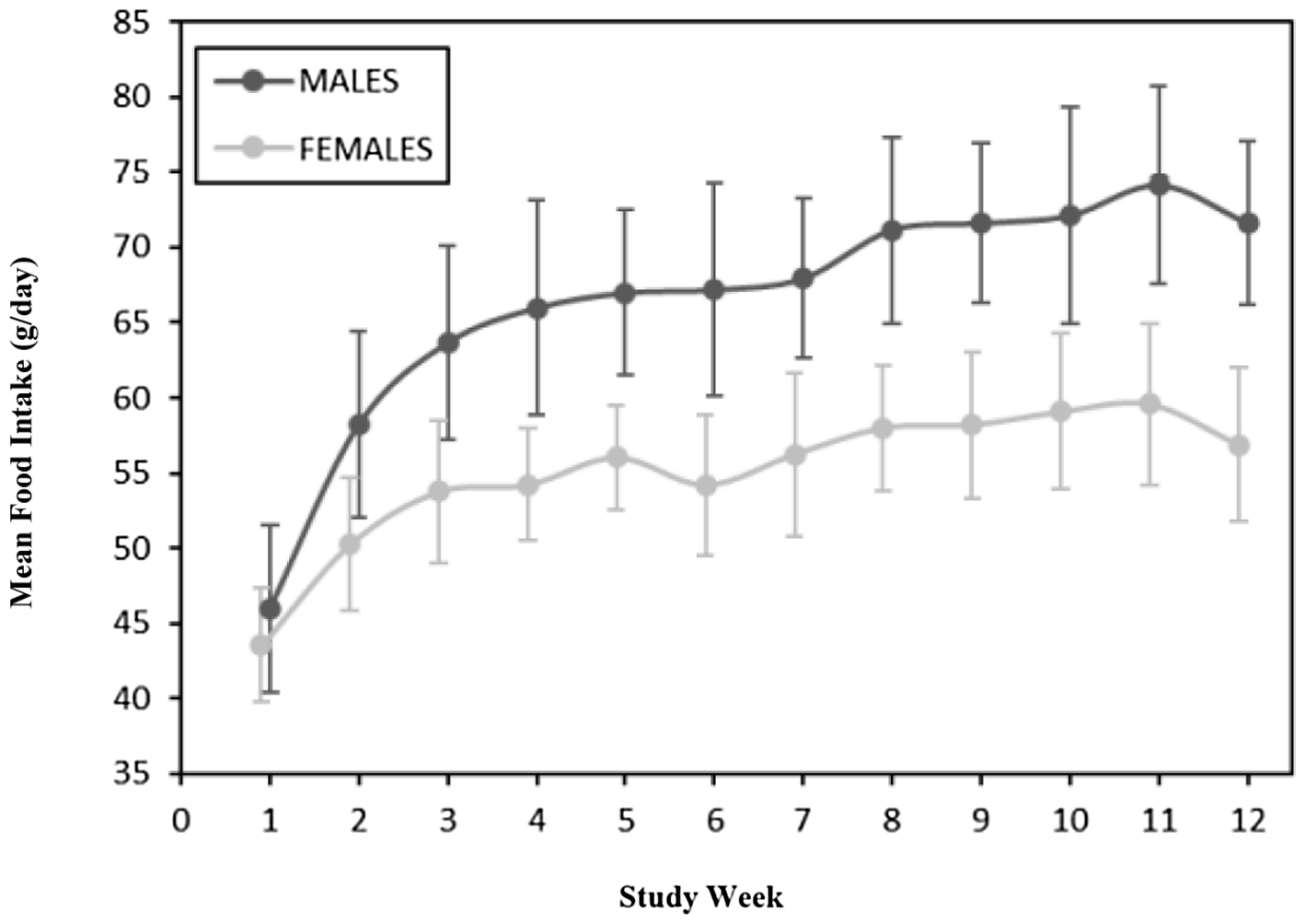
Average food intake (g/day) for males and females.

**Figure 5:**
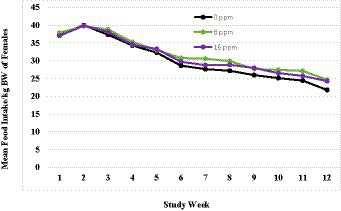
Mean daily food intake in grams per kilogram of body weight calculated for females.

**Figure 6:**
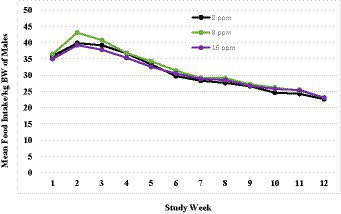
Mean daily food intake in grams per kilogram of body weight calculated for males.

**Figure 7:**
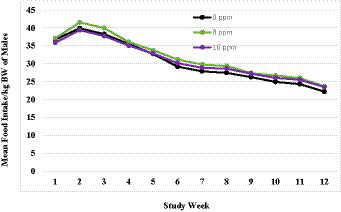
Mean daily food intake in grams per kilogram of body weight calculated for the three diet groups.

A summary of weekly body weights can be found in Figures 2, 3. Figure 2 shows the differences in weight gain between sexes, and Figure 3 shows the differences in weight gain between feeding groups. Males weighed more than females, and a sex-by-age interaction was observed due to the differences between sexes increasing with increasing age. Statistical analysis found significant effects of sex (*p* < 0.001) and test week (*p* < 0.001). There was also a statistically significant correlation between test week and sex regarding weight gain. However, there was no significant difference in weights or weight gain among diet groups (*p* = 0.83), nor was there a significant correlation between diet and test week (*p* = 0.45). Mean body weight increased each week across all test groups. The average body weight gain (kg) throughout the study was 1.75 ± 0.11 for the 0 ppm diet group, 1.78 ± 0.12 kg for the 8 ppm group, and 1.71 ± 0.13 for the 16 ppm group. Body condition scores were not statistically analyzed, as food intake was adjusted throughout the study to ensure all kittens maintained a BCS score of 5 on a scale of 1–9. Although a subset of subjects had a BCS of 4/9 upon arrival, by week 6 all subjects had a BCS of 5/9. Throughout the study, none of the kittens had a BCS of less than 4/9 or a BCS greater than 6/9, and all kittens finished the study with a BCS of 5/9. The summary of average daily food intake can be found in Figure 4 for both sexes, Figure 5 for males, and Figure 6 for females. Figure 7 depicts the mean daily food intake in grams *per* kilogram of body weight calculated for males and females combined. Mean daily food intake, which was calculated by dividing the mean daily intake by the mean bodyweight, in grams *per* kilogram of bodyweight (g/kg) was calculated for each week of the study. The food intake was comparable across diet groups for males and females, and when sexes were combined *per* kg bodyweight. There were statistically significant main effects of sex (*p* < 0.001) and test week (*p* < 0.001) and a statistically significant interaction between test week and sex (*p* < 0.001). However, there was no effect on food intake when both sexes were combined. There was also no significant interaction between diet and test week (*p* = 0.91).

### Clinical evaluations

All kittens remained in good health through the study. The veterinary examinations did not reveal any clinical abnormalities that would affect the kittens’ overall health or participation in the study. Upon arrival to the test facility, several kittens exhibited ocular and nasal discharge, which were attributed to the stress of transport as they resolved without intervention. Abnormal health observations were minor and unrelated to dietary treatment. No abnormalities in the kittens’ behavior or social interactions were observed during the course of the study.

### Clinical chemistry

Clinical chemistry parameters are described in Table 2, which were analyzed on Day 84 of the study. Minimum AAFCO animal feeding test standards (47) were met in all groups for albumin, with a group average > 2.7 g/dL and with no individual <2.4 g/dL. The clinical chemistry data were typical of healthy, growing kittens and there were no statistically significant differences between any of the diet groups (Table 2). Overall, all clinical chemistry parameters, with the exception of those described below, were within reference ranges.

**Table 2:** Clinical chemistry values (mean ± SD) for both male and female kittens on Day 84 of study.

**Males**	**0 ppm AFD1**	**8 ppm AFD1**	**16 ppm RAFD1**	***p*-value**	***Laboratory Reference range (Adult)***
Alkaline Phosphatase (ALP, U/L)	75.44 ± 5.042	74.78 ± 5.489	82.00 ± 8.232	0.683	12 - 59
Aspartate Aminotransferase (AST, U/L)	21.22 ± 1.714	19.44 ± 1.168	19.33 ± 0.90	0.526	16 - 67
Alanine Aminotransferase (ALP, U/L)	45.00 ± 4.26	45.56 ± 3.35	43.22 ± 1.26	0.866	27 - 158
Albumin (ALB, g/dL)	3.61 ± 0.035	3.57 ± 0.071	3.58 ± 0.365	0.810	2.6 - 3.9
Bilirubin (mg/dL)	0.178 ± 0.015	0.189 ± 0.011	0.189 ± 0.011	0.768	0.0 - 0.3
Total Protein (g/dL)	6.39 ± 0.082	6.31 ± 0.072	6.42 ± 0.092	0.627	6.3 - 8.8
Globulin (GLOB, g/dL)	2.778 ± 0.079	2.744 ± 0.033	2.844 ± 0.010	0.659	3.0 - 5.9
Blood Urea Nitrogen (BUN, mg/dL)	25.78 ± 0.954	26.22 ± 1.187	26.56 ± 0.580	0.843	16 - 37
Creatinine (mg/dL)	1.022 ± 0.036	0.989 ± 0.0206	0.967 ± 0.037	0.482	0.9 -2.5
Cholesterol (mg/dL)	148.7 ± 6.782	152.4 ± 7.879	162.3 ± 6.285	0.395	91 - 305
Glucose (mg/dL)	96.00 ± 2.134	95.33 ± 2.010	91.67 ± 1.624	0.253	72 - 175
Calcium (mg/dL)	10.47 ± 0.085	10.56 ± 0.060	10.41 ± 0.081	0.378	8.6 - 10.6
Phosphorous (mg/dL)	8.356 ± 0.186	8.156 ± 0.111	8.233 ± 0.199	0.707	2.9 - 6.3
Chloride (nmol/L)	115.89 ± 0.484	115.22 ± 0.323	115.67 ± 0.29	0.453	114 -126
Potassium (nmol/L)	4.844 ± 0.121	4.944 ± 0.076	4.977 ± 0.145	0.719	3.7 -5.2
ALB/GLOB (ratio)	1.31 ± 0.035	1.30 ± 0.04	1.27 ± 0.05	0.746	0.5 - 1.2
Sodium (mmol/L)	154.22 ± 20.641	153.56 ± 0.294	153.90 ± 0.309	0.577	147 - 157
BUN/Creatinine (ratio)	25.244 ± 0.556	30.078 ± 3.000	27.744 ± 1.066	0.208	

Rows in red denote values outside of the provided reference range.

The alkaline phosphatase (ALP) levels were above the reference range for adult cats (Table 3). Although the values were outside of the adult-based reference range used by the laboratory, all values were within age-specific reference ranges at both time points (48).

**Table 3:** Hematology values (mean ± SD) for both male and female kittens on Day 84 of study.

	Group Means and Standard Errors				
**Males**	**0 ppm AFD1**	**8 ppm AFD1**	**16 ppm AFD1**	***p*-value**	***Reference Range***
Hemolysis	4 high	1 high	All normal		
Lipemia	All normal	All normal	All normal		
White Blood Cells (WBC, K/ μl)	18.82 ± 1.07	16.57 ± 0.86	15.07 ± 1	0.04*	3.9 - 19
Red Blood Cells (RBC, M/ μl)	9.70 ± 0.21	9.28 ± 0.31	9.82 ± 0.17	0.25	7.12 - 11.46
Hemoglobin (HGB, g/dL)	12.71 ± 0.32	11.79 ± 0.30	12.18 ± 0.29	0.12	0.3 - 16.2
Hematocrit (HCT, %)	39.02 ± 1.12	36.04 ± 1.01	37.07 ± 1.26	0.19	28.2 - 52.7
Mean Corpuscular Volume (MCV, fL)	40.00 ± 1.04	38.89 ± 0.841	37.78 ± 0.81	0.24	39 - 56
Mean Corpuscular Hemoglobin (MCH, pg)	13.09 ± 0.19	12.74 ± 0.27	12.41 ± 0.16	1	12.6 - 16.5
Mean Corpuscular Hemoglobin Concentration (MCHC, g/dL)	32.62 ± 0.45	32.73 ± 0.4	32.97 ± 0.5	0.86	28.5 - 37.8
Platelet (K/μl)	379.44 ± 62.20	500.67 ± 34.90	477.78 ± 24.69	0.13	155 - 641
Reticulocytes (%)	0.17 ± 0.04	0.17 ± 0.02	0.14 ± 0.02	0.78	
Absolute Reticulocytes (K/μl)	None	None	None		3 - 50
Nucleated RBC (/100 WBC)	None	None	None		
Neutrophil (/μl)	7620.8 ± 811.5	6855.6 ± 469.4	6852.11 ± 904.5	0.90	2620 - 15170
Lymphocyte (/μl)	9914.89 ± 949.7	8387.7 ± 655.6	6826.33 ± 458.4	0.019*	850 - 5850
Monocyte (/μl)	600.44 ± 75.42	555.67 ± 46.04	499.89 ± 39.50	0.46	40-530
Eosinophil (/μl)	1025.67 ± 147.92	753.56 ± 74.12	874.89 ± 74.09	0.21	90-2180
Basophil (/μl)	20.33 ± 1.34	14.89 ± 2.04	14.11 ± 2.02	0.05*	0-100

Rows in red denote values outside of the provided reference range. *Statistically significant difference (*p* ≤ 0.05).

None of the kittens exhibited hypergammaglobulinemia, but globulin levels for the majority of the kittens were below the lab-reported reference range based on adult cats (Table 3); as a result, the albumin:globulin values were above reference range. A kitten-specific reference range for globulin was not available. However, all but one kitten demonstrated increased globulin levels at Day 84 compared to Day −6, and all kittens remained healthy with no clinical or laboratory indications of infection or immune compromise. These variations did not follow a specific AFD1 level-related progression and were not consistent between the male and female groups.

Phosphorus levels were above the provided reference range but within kitten-specific published reference ranges.

### Hematology

The results of hematological analysis are shown in Table 3. Minimum AAFCO animal feeding test standards were met for the predetermined parameters of interest (47), which include hemoglobin group average (>10.0 g/dL0, with no individual level being 8.0 g/dL) and hematocrit (HCT, PCV) group average (>30%, with no individual value being <26%). The majority of hematology parameters measured were unremarkable and within reference or expected ranges. Through the aforementioned statistical analyses, three parameters were found to have statistically significant group differences: white blood cell (WBC), lymphocyte, and basophil counts. The corresponding value of *p*s were 0.04, 0.02, and 0.05, respectively (Table 3). These parameters were then assessed using post-hoc analysis and compared utilizing the Tukey’s multiple comparison test.

For total WBC, the mean count was significantly higher in the 0 ppm group (control) than the 16 ppm diet group (*p* = 0.03), but not the 8 ppm diet group (*p* = 0.25; Table 4). There were also no significant differences between the 8 ppm and 16 ppm diet groups (*p* = 0.53; Table 4).

**Table 4:** *Post-hoc* Tukey’s analysis comparing WBC (K/μL) levels of the three diet groups.

Group	0 ppm AFD1 Mean = 18.82	8 ppm AFD1 Mean = 16.57	16 ppm AFD1 Mean = 15.07
0 ppm	N/A	0.25	0.03*
8 ppm	0.25	N/A	0.53
16 ppm	0.03*	0.53	N/A

*Statistically significant difference (*p* ≤ 0.05).

The mean lymphocyte count was significantly higher in the control group than the 16 ppm group (*p* = 0.02), but not the 8 ppm group (*p* = 0.31; Table 5). There were also no significant differences between the 8 and 16 ppm diet groups (*p* = 0.29; Table 5). For basophils, the mean count did not significantly differ between the control and 16 ppm groups (*p* = 0.06), between control and 8 ppm diet groups (*p* = 0.11; Table 6), nor between 8 and 16 ppm groups (*p* = 0.95; Table 6). Despite the statistical significance between groups, the lymphocyte and basophil counts remained within age-specific reference ranges (49). The following parameters were not compared statistically because of the absence of normality: reticulocytes, absolute reticulocytes, and nucleated red blood cells (RBC).

**Table 5:** *Post-hoc* Tukey’s analysis comparing lymphocyte (/μL) levels of the three diet groups.

Group	0 ppm AFD1 Mean = 9,914.9	8 ppm AFD1 Mean = 8,387.7	16 ppm AFD1 Mean = 6,826.3
0 ppm	N/A	0.31	0.01*
8 ppm	0.31	N/A	0.29
16 ppm	0.01*	0.29	N/A

*Statistically significant difference (*p* ≤ 0.05).

**Table 6:** *Post-hoc* Tukey’s analysis comparing basophil (/μL) levels of the three diet groups.

Group	0 ppm AFD1 Mean = 9,914.9	8 ppm AFD1 Mean = 8,387.7	16 ppm AFD1 Mean = 6,826.3
0 ppm	N/A	0.11	0.06
8 ppm	0.11	N/A	0.95
16 ppm	0.06	0.95	N/A

Mild lymphocytosis and monocytosis were observed in subjects from all diet groups. These variations did not follow a specific AFD1 level-related progression and were not consistent between the male and female groups.

### Taurine

The results of plasma taurine analysis are shown in Table 7. Analysis demonstrated that sex (*p* = 0.07) and test groups (*p* = 0.94) had no significant differences in serum taurine levels. Taurine levels were outside reference ranges for adult cats (50, 51), but plasma taurine levels in kittens have been reported above adult cat reference range (80–120 nmoL/mL) in multiple publications (52–54).

**Table 7:** Summary of blood taurine levels (nmol/ml)* at Day 84.

Group	Number	Mean (nmol/ml)	Minimum (nmol/ml)	Maximum (nmol/ml)	Standard deviation (nmol/ml)
**Summary: test group (combined males and females)**					
0 ppm	9	487.22	394	609	68.81
8 ppm	9	495.56	301	642	110.60
16 ppm	9	508.33	373	716	104.65
**Summary: test group (females)**					
0 ppm	5	503.60	401	609	75.43
8 ppm	4	436.25	301	532	114.73
16 ppm	4	433.25	373	493	64.03
**Summary: test group (males)**					
0 ppm	4	466.75	394	549	63.52
8 ppm	5	543.00	435	642	90.88
16 ppm	5	568.40	469	716	93.16
**Summary: sex (all groups combined)**					
Females	13	461.23	301	609	86.20
Males	14	530.29	394	716	89.47

*Adult reference range 80–120 nmoL/mL.

## Discussion

The present study evaluated the effect of an AFD1 ingredient for pet food on growth performance in kittens on overall health, growth performance, food consumption, clinical chemistry and hematological parameters, and taurine levels in kittens when compared to a commercially available, complete and balanced control diet that also served as the base for the test diet. No biologically meaningful alterations were observed in the clinical chemistry, hematology, coagulation, or clinical parameters that that could be attributed to dietary AFD1 provided to kittens for 12 weeks at the levels tested.

Previously, Matulka et al. (33) demonstrated that a diet coated with a chicken egg product containing AFD1 fed to adult male and female cats was well tolerated and did not exhibit any clinically relevant effects on food consumption, weight gain, clinical chemistry, or hematology parameters. The *in vitro* genotoxicity studies conducted by Matulka et al., consistent with standard safety testing required by the US Food and Drug Administration (FDA), demonstrated that that AFD1 ingredient does not induce mutagenic effects or chromosomal aberrations.

The data from this study were compared to the criteria set forth by the 2020 Official Publication produced by the AAFCO (47), apart from taurine data that was reviewed by the attending veterinarian and compared to published reference ranges. The egg product containing AFD1 was safe and well-tolerated in 8–21-week-old kittens according to the AAFCO standards. There is a certain amount of intra- and inter-individual variation with analysis of hematological and clinical endpoints; therefore, a systemic assessment of the data is necessary to determine the overall health of the subjects. No animals in any of the feeding groups of either sex exhibited clinical or pathological signs of nutritional deficiency or excess as determined by hematological and clinical chemistry analysis, taurine analysis, veterinary examination, food consumption, body weight and condition, health observations, and lack of adverse effects.

The results of bodyweight analysis of adult cats in Matulka et al. (33) were similar to the results observed in this study. Males weighed significantly more than females; however, there were no significant differences in body weights between the control group or any group receiving AFD1 at any dose, for both sexes. Overall, as in this current study with kittens, adult male cats consumed more than female cats (33).

Significant differences in kittens consuming the 16 ppm AFD1 diet (as compared to the 0 ppm diet) were observed in three of the tested hematological parameters (WBC, lymphocyte, and basophil count), with only WBC and lymphocyte counts exhibiting statistical significance after post-hoc Tukey’s multiple comparison test. However, the WBC, lymphocyte, and basophil counts were within publicly available kitten-specific reference ranges (47, 49, 55). Moreover, none of the kittens within the study presented with physical or behavioral abnormalities, adverse events, or clinical conditions that would be associated with clinically relevant changes in these hematological parameters. The variations with the hematological parameters were associated with biological variation and normal handling, and not attributable to AFD1 administration. Similar to this current study, analysis of hematology parameters in the study with adult cats by Matulka et al. (33) revealed that test groups had generally similar values as compared to the control group. Overall, AFD1 had no clinically relevant effect on hematological parameters of kittens or adult cats as determined by this study and Matulka et al., respectively (33).

Clinical chemistry results reported in kittens were comparable to those obtained for adult cats in the study by Matulka et al. (33). Overall, all measured clinical chemistry values were within ranges expected for clinically healthy cats for both sexes. ALP levels in kittens were above the adult reference range; however, this is expected in growing kittens since ALP is associated with bone growth and development. Furthermore, no significant difference in ALP was found between the groups in this study. Despite the lack of an age-specific reference range for globulin levels, there was no group effect identified. AFD1 had no clinically relevant effect on clinical chemistry or taurine levels in both kittens and adults based on the results of this study and Matulka et al. (33).

No abnormalities in behavior or social interaction were observed in this study or in adult cats in the previous study by Matulka et al. (33). Although the role of Fel d 1 has not been definitively established, proposed roles include pheromone/chemical signaling (11–18). Therefore, completely eliminating Fel d 1 could theoretically alter social interactions, although this has not been confirmed. Fel d 1 production varies widely among cats, with some cats producing many times the level of others and may even vary widely within the same cat over the course of the year (7). The AFD1 approach utilized in this study significantly reduces Fel d 1 but does not eliminate it. Therefore, variable levels of Fel d 1 remain available for the cat’s possible physiological needs while the total allergen level is reduced to benefit sensitized human individuals.

Satyaraj et al. (41) found that a complete and balanced feline dry diet coated with an egg product containing AFD1 significantly reduced active (allergenic) Fel d 1 saliva concentrations beginning in the third week of consumption. Satyaraj et al. (56) also conducted a 12-week study in 105 cats to evaluate the efficacy of the diet in reducing active Fel d 1 on cats’ hair. The authors reported that the diet reduced active Fel d 1 (with an average decrease of 47%, ranging from a 33–71% decrease versus baseline, and 50 and 86% of the cats exhibiting ≥50% and ≥ 30% reduction in AFD1, respectively), was well tolerated, and had no adverse effects that required clinical intervention or removal of subjects from the study (56).

The approach described in this study and previously published studies (33, 41–43, 56) addresses salivary Fel d 1. Other sources of Fel d 1 include the sebaceous, lacrimal, and anal glands (3). The relative contributions of each Fel d 1 source to total Fel d 1 on cats’ hair and eventually into the environment have not been identified, although the salivary and sebaceous glands are generally considered to contribute the largest portions (3). Satyaraj et al. (56) documented a significant reduction in Fel d 1 levels on cats’ hair associated with this salivary Fel d 1-targeted approach, supporting the large contribution of salivary Fel d 1 to total Fel d 1. Residual unbound anti-Fel d 1 IgY in the cats’ saliva could potentially neutralize Fel d 1 from other sources as the saliva is dispersed during grooming, but this has not been confirmed.

The results of this study corroborate the conclusion that the egg product containing AFD1 is well tolerated in growing kittens. The AFD1-coated diet with a control or AFD1-containing top-dress was successful for promoting normal growth and development in 8–21-week-old kittens in the absence of adverse health or behavioral effects or clinical outcomes. Based on the current and previous studies (33, 41, 56), AFD1 significantly reduces the amount of allergenic Fel d 1 in cats’ saliva and on their hair without adversely affecting the cat’s overall health. This approach does not alter Fel d 1 production by cats, but instead neutralizes the allergen after it is secreted. AFD1 antibody incorporation represents a novel, effective, and safe method for reducing Fel d 1. As part of a comprehensive allergen management plan, this approach can help reduce cat allergens while keeping cats in loving homes.

## Data availability statement

The original contributions presented in the study are included in the article/supplementary material, further inquiries can be directed to the corresponding author.

## Ethics statement

The animal study was approved by CanCog (Vivocore Internal Animal Care Committee). The study was conducted in accordance with the local legislation and institutional requirements.

## Author contributions

EH: Writing – original draft, Data curation, Conceptualization. RM: Writing – original draft. LC-S: Writing – review & editing. KM: Writing – review & editing.
